# Duodenal Atresia Associated with Apple Peel Atresia and Situs Inversus Abdominus: A Case Report

**DOI:** 10.21699/jns.v5i4.420

**Published:** 2016-10-10

**Authors:** H. Ben Hamida, R. Hadj Salem, K. Ben Ameur, A. Rassas, FZ. Chioukh, R. Sakka, N. Kechiche, M. Bizid, L. Sahnoun, K. Monastiri

**Affiliations:** 1 Intensive Care and Neonatal Medicine Department, Teaching Hospital of Monastir, University of Monastir, Tunisia; 2 Pediatric Surgery Department, Teaching Hospital of Monastir, University of Monastir, Tunisia; 3 Faculty de Medicine, University of Monastir, Tunisia

**Keywords:** Duodenal atresia, Apple peel atresia, Agenesis of superior mesenteric artery, Situs inversus

## Abstract

Duodenal atresia is rarely associated with situs inversus abdominus. We report a case of duodenal atresia associated with small bowel atresia of apple peel type and situs inversus abdominus.

## CASE REPORT

A 3-kg baby boy was born by spontaneous vaginal delivery at 34 weeks of gestation to a 37-year-old mother, G6P4A2, whose pregnancy was complicated by diabetes mellitus and toxemia, treated respectively by insulin and anti-hypertensive drugs prior to delivery. Prenatal ultrasonography reports were normal. At birth, the baby was admitted to the neonatal intensive care unit for respiratory distress related to an early neonatal infection. On the second day of life, bilious vomiting and meconium emission delay were noticed. An abdominal X-ray revealed double bubble sign with no air distally and the stomach was localized in the right side of the abdomen. An upper contrast study confirmed the diagnosis of complete duodenal obstruction and right sided stomach (Fig.1). Abdominal ultrasonography revealed liver located in the left upper quadrant of the abdomen and a spleen in the right one. Surgical exploration showed atresia of the distal portion of duodenum and of the proximal jejunum, multiple atresia of the remaining small bowel wrapping around its blood supply in a spiral manner resembling an apple peel, mesenteric defect, agenesis of the superior mesenteric artery and abdominal situs inversus (Fig.2). Nothing could be done peroperatively. The baby was kept perfused on a central venous catheter and died after 32 days. 

**Figure:**
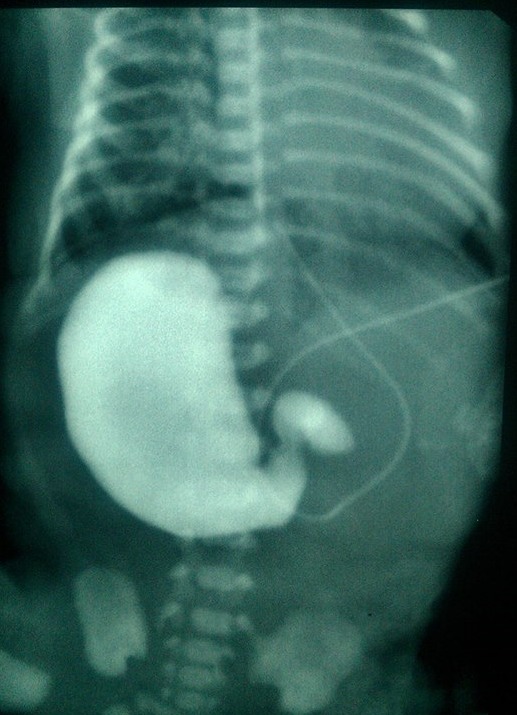
Figure 1: Contrast study showing right sided stomach and duodenal obstruction.

**Figure:**
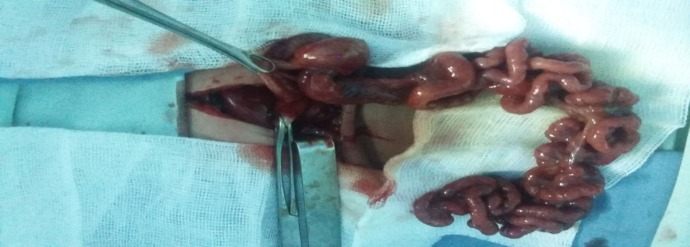
Figure 2: Apple peel configuration in addition to duodenal atresia. Multiple atresias were also present throughout length of small bowel.

## DISCUSSION

Duodenal atresia is a relatively common anomaly with an estimated incidence of 1 in 3000 to 1 in 5000 live births [1]. It is also common for duodenal atresia to be associated with other anomalies mainly congenital heart disease and Down's syndrome [1]. Our case had duodenal atresia, multiple atresias including apple peel type, and situs inversus abdominus. The association of these two types of intestinal atresia is extremely rare and only five cases were reported before [2]. Our case is unique as it has additional association with situs inversus abdominus which is not reported earlier. The management of these patients is complex. The difficulty lies in the treatment of apple peel atresias that continues to have a serious prognosis with significant morbidity and mortality [3]. Our baby was considered inoperable because of the extent of multiple intestinal atresias that make the attempt of multiple primary anastomoses difficult. Rich et al [3] reported a successfully treated apple peel syndrome with a total of six primary small bowel anastomoses. Other procedures were described to treat multiple intestinal atresias: silicone stenting as a method to preserve bowel length and enterostomy [4]. In our case, the decision was taken not to intervene further in consultation with parents based on poor prognosis.

## Footnotes

**Source of Support:** Nil

**Conflict of Interest:** Nil
